# 6-Chloro-2-(thio­phen-2-yl)-1-[(thio­phen-2-yl)meth­yl]-1*H*-benzimidazole

**DOI:** 10.1107/S1600536813011124

**Published:** 2013-04-30

**Authors:** David K. Geiger, Michael R. Nellist

**Affiliations:** a Department of Chemistry, State University of New York-College at Geneseo, 1 College Circle, Geneseo, NY 14454, USA

## Abstract

The title compound, C_16_H_11_ClN_2_S_2_, co-crystallizes with a small amount of the 5-chloro- isomer. The ratio of 6-chloro- to 5-chloro- isomers is 0.969 (2):0.031 (2). One thio­phen-2-yl substitutent displays rotational disorder with 80.6 (4)% of the mol­ecules exhibiting the major orientation. In the crystal, weak C—H⋯N and C—H⋯S hydrogen-bonding inter­actions result in chains of mol­ecules parallel to [001].

## Related literature

For the structure of 2-(thio­phen-2-yl)-1-(thio­phen-2-ylmeth­yl)-1*H*-benzimidazole, see: Geiger *et al.* (2012 ▶). For the structure of the 5-bromo analogue, see: Geiger & Destefano (2012 ▶).
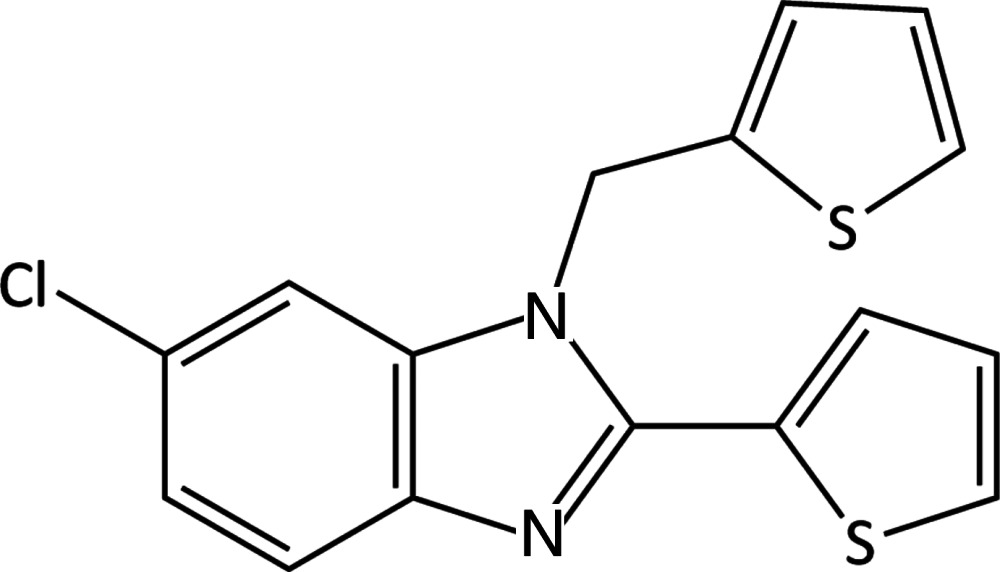


## Experimental

### 

#### Crystal data

C_16_H_11_ClN_2_S_2_
*M* *_r_* = 330.84
Monoclinic, 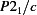

*a* = 15.465 (3) Å
*b* = 6.3578 (10) Å
*c* = 15.634 (3) Å
β = 103.687 (5)°
*V* = 1493.5 (4) Å^3^
*Z* = 4
Mo *K*α radiation
μ = 0.53 mm^−1^
*T* = 200 K
0.80 × 0.60 × 0.20 mm

#### Data collection

Bruker SMART X2S CCD diffractometer
Absorption correction: multi-scan (*SADABS*; Bruker, 2010 ▶) *T*_min_ = 0.62, *T*_max_ = 0.90
23773 measured reflections
2665 independent reflections
2184 reflections with *I* > 2σ(*I*)
*R*_int_ = 0.088

#### Refinement

*R*[*F* ^2^ > 2σ(*F* ^2^)] = 0.056
*wR*(*F* ^2^) = 0.148
*S* = 1.11
2665 reflections
210 parameters
38 restraints
H-atom parameters constrained
Δρ_max_ = 0.49 e Å^−3^
Δρ_min_ = −0.46 e Å^−3^

### 

Data collection: *APEX2* (Bruker, 2010 ▶); cell refinement: *SAINT* (Bruker, 2010 ▶); data reduction: *SAINT*; program(s) used to solve structure: *SHELXS97* (Sheldrick, 2008 ▶); program(s) used to refine structure: *SHELXL97* (Sheldrick, 2008 ▶); molecular graphics: *PLATON* (Spek, 2009 ▶) and *Mercury* (Macrae *et al.*, 2008 ▶); software used to prepare material for publication: *publCIF* (Westrip, 2010 ▶).

## Supplementary Material

Crystal structure: contains datablock(s) global, I. DOI: 10.1107/S1600536813011124/pv2630sup1.cif

Structure factors: contains datablock(s) I. DOI: 10.1107/S1600536813011124/pv2630Isup2.hkl

Supplementary material file. DOI: 10.1107/S1600536813011124/pv2630Isup3.mol

Supplementary material file. DOI: 10.1107/S1600536813011124/pv2630Isup4.cml

Additional supplementary materials:  crystallographic information; 3D view; checkCIF report

## Figures and Tables

**Table 1:** Hydrogen-bond geometry (Å, °)

*D*—H⋯*A*	*D*—H	H⋯*A*	*D*⋯*A*	*D*—H⋯*A*
C16—H16⋯N2^i^	0.95	2.62	3.369 (4)	136
C12—H12*B*⋯N2^ii^	0.99	2.69	3.654 (4)	165
C12—H12*B*⋯S1^ii^	0.99	2.99	3.599 (4)	120

Symmetry codes: (i) 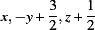; (ii) 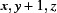.

**Fig. 1.**
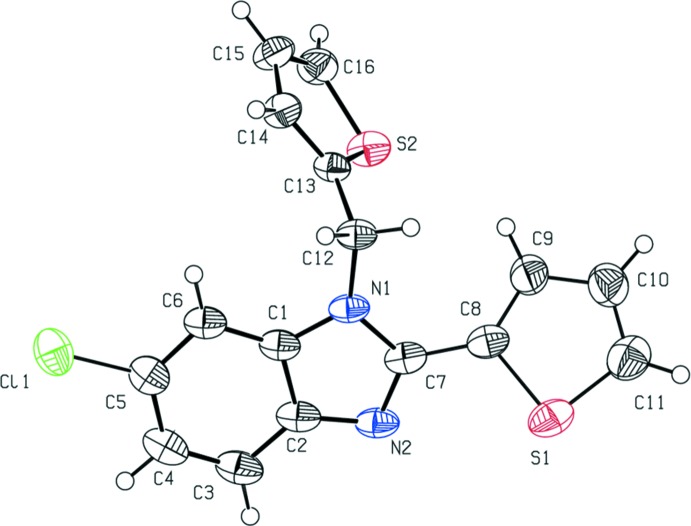
Perspective view of the title compound. Thermal parameters are drawn at the 50% probability level. Only major contributors to the disorder model are shown.

**Fig. 2.**
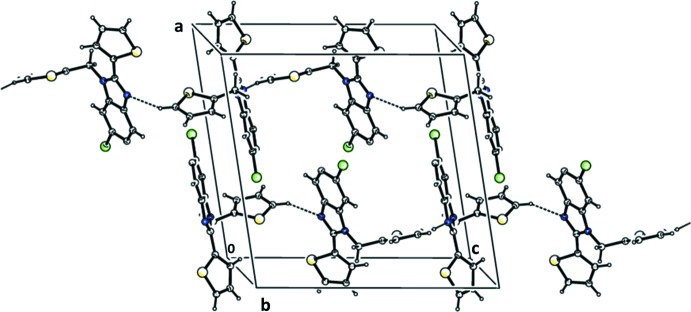
Packing diagram showing the chains along [0 0 1] formed by weak C—H···N H-bonding interactions. Only the major contributor to the disorder model is shown.

## supplementary crystallographic information

### Comment

The bond distances and angles in the title compound (Fig. 1) agree very well with the corresponding bond distances and angles reported in closely related compounds (Geiger *et al.*, 2012; Geiger & Destefano, 2012). Crystallization of the title compound occurs with 3.1 (2)% of the sites occupied by the 5-chloro isomer. The 2-thiophene substituent is rotationally disordered with the major component having a refined occupancy of 81.0 (4)%. The benzimidazole moiety is planar (r. m. s. deviation = 0.0079 Å) with the largest deviation being for C1, which is 0.014 (3) Å out of the plane. The 2-thiophene substitutents are canted 33.2 (2)% from the benzimidazole plane for the major disorder component and 27 (1)% for the minor component.

Molecules are joined by weak C16—H16···N2 H-bonding resulting in chains parallel to [0 0 1]. The H16···N2 distance is 2.62 Å and the C16—H16···N2 angle is 136°. Weak C12—H12B···N2 and C12—H12B···S1 H-bonding joins the chains of molecules parallel to [0 0 1] (Tab. 1 & Fig. 2).

### Experimental

1,2-Diamine-4-chlorobenzene (6.3 mmol, 0.90 g) was dissolved in 30 ml ethanol under nitrogen. Two equivalents of 2-thiophenecarboxaldehyde (1.3 ml) was added dropwise. After three days, the solvent was removed under reduced pressure and the crude product was chromatographed (silica gel) using a mixture of 30% hexane in ethyl acetate. The first fraction produced hexagonal shaped crystals (1) and the second fraction produced needle-shaped crystals (2) on slow evaporation. Crystals from the first fraction were used for X-ray diffraction experiments. The overall yield was 59%.

### Refinement

All hydrogen atoms were observed in difference fourier maps. The H atoms were refined using a riding model with a C—H distance of 0.99 Å for the methylene carbon atoms and 0.95 Å for the phenyl and thiophene carbon atoms. All C—H hydrogen atom thermal parameters were set using the approximation *U*_iso_ = 1.2*U*_eq_. The Cl and H atoms of the major and minor co-crystallization components were modeled as a disorder involving two parts, each containing a chlorine atom and a hydrogen atom. The major component refined to a site occupancy of 0.969 (2).

In addition, the 2-thiophene substituent is rotationally disordered. A model was developed in which the minor component of the thiophene ring was defined using the metrics of the major component as a guide. The disordered five-member rings were constrained to planarity using FLAT. Corresponding bond distances of the minor component and major component were set equal using SAME and corresponding thermal parameters were held the same using EADP. All atoms were refined anisotropically with hydrogen atoms in calculated positions using a riding model. With these constraints, the site occupancy of the major component refined to 0.806 (4).

### Crystal data

**Table:**

C_16_H_11_ClN_2_S_2_	*F*(000) = 680
*M**_r_* = 330.84	*D*_x_ = 1.471 Mg m^−^^3^
Monoclinic, *P*2_1_/*c*	Mo *K*α radiation, λ = 0.71073 Å
*a* = 15.465 (3) Å	Cell parameters from 7129 reflections
*b* = 6.3578 (10) Å	θ = 2.7–25.0°
*c* = 15.634 (3) Å	µ = 0.53 mm^−^^1^
β = 103.687 (5)°	*T* = 200 K
*V* = 1493.5 (4) Å^3^	Plate, colourless
*Z* = 4	0.80 × 0.60 × 0.20 mm

### Data collection

**Table:**

Bruker SMART X2S CCD diffractometer	2665 independent reflections
Radiation source: XOS X-beam microfocus source	2184 reflections with *I* > 2σ(*I*)
Doubly curved silicon crystal monochromator	*R*_int_ = 0.088
Detector resolution: 8.3330 pixels mm^-1^	θ_max_ = 25.4°, θ_min_ = 2.7°
ω scans	*h* = −18→18
Absorption correction: multi-scan (*SADABS*; Bruker, 2010)	*k* = −7→7
*T*_min_ = 0.62, *T*_max_ = 0.90	*l* = −18→18
23773 measured reflections	

### Refinement

**Table:**

Refinement on *F*^2^	Primary atom site location: structure-invariant direct methods
Least-squares matrix: full	Secondary atom site location: difference Fourier map
*R*[*F*^2^ > 2σ(*F*^2^)] = 0.056	Hydrogen site location: inferred from neighbouring sites
*wR*(*F*^2^) = 0.148	H-atom parameters constrained
*S* = 1.11	*w* = 1/[σ^2^(*F*_o_^2^) + (0.0645*P*)^2^ + 1.4022*P*] where *P* = (*F*_o_^2^ + 2*F*_c_^2^)/3
2665 reflections	(Δ/σ)_max_ < 0.001
210 parameters	Δρ_max_ = 0.49 e Å^−^^3^
38 restraints	Δρ_min_ = −0.46 e Å^−^^3^

### Special details

**Table:**

Experimental. ^1^H NMR spectrum (CDCl_3_, 400 MHz, p.p.m.): 7.71 (1 H, *d*), 7.53 (1 H, *d*), 7.48 (1 H, *d*), 7.34 (1 H, *s*), 7.28 (2 H, *m*), 7.17 (1 H, *t*), 6.96 (1 H, *t*), 6.91 (1 H, *d*), 5.60 (2 H, *s*).
Geometry. All e.s.d.'s (except the e.s.d. in the dihedral angle between two l.s. planes) are estimated using the full covariance matrix. The cell e.s.d.'s are taken into account individually in the estimation of e.s.d.'s in distances, angles and torsion angles; correlations between e.s.d.'s in cell parameters are only used when they are defined by crystal symmetry. An approximate (isotropic) treatment of cell e.s.d.'s is used for estimating e.s.d.'s involving l.s. planes.
Refinement. Refinement of *F*^2^ against ALL reflections. The weighted *R*-factor *wR* and goodness of fit *S* are based on *F*^2^, conventional *R*-factors *R* are based on *F*, with *F* set to zero for negative *F*^2^. The threshold expression of *F*^2^ > σ(*F*^2^) is used only for calculating *R*-factors(gt) *etc*. and is not relevant to the choice of reflections for refinement. *R*-factors based on *F*^2^ are statistically about twice as large as those based on *F*, and *R*- factors based on ALL data will be even larger.

### Fractional atomic coordinates and isotropic or equivalent isotropic displacement parameters (Å^2^)

**Table:**

	*x*	*y*	*z*	*U*_iso_*/*U*_eq_	Occ. (<1)
Cl1	0.55843 (7)	1.26049 (17)	0.40613 (7)	0.0561 (3)	0.969 (2)
Cl01	0.5861 (12)	0.817 (5)	0.327 (2)	0.0561 (3)	0.031 (2)
S1	0.06013 (11)	0.5424 (3)	0.29173 (10)	0.0530 (5)	0.806 (4)
C8	0.1134 (4)	0.7175 (17)	0.3728 (6)	0.0402 (8)	0.806 (4)
C9	0.0602 (5)	0.7532 (12)	0.4316 (6)	0.054 (2)	0.806 (4)
H9	0.0787	0.8349	0.4836	0.065*	0.806 (4)
C10	−0.0230 (4)	0.6567 (13)	0.4065 (5)	0.0593 (16)	0.806 (4)
H10	−0.0684	0.6749	0.4375	0.071*	0.806 (4)
C11	−0.0327 (4)	0.5363 (12)	0.3347 (5)	0.0610 (18)	0.806 (4)
H11	−0.0847	0.4567	0.3103	0.073*	0.806 (4)
S101	0.0553 (7)	0.8069 (15)	0.4406 (7)	0.0530 (5)	0.194 (4)
C108	0.1165 (15)	0.718 (7)	0.370 (3)	0.0402 (8)	0.194 (4)
C109	0.0711 (18)	0.573 (5)	0.3138 (19)	0.054 (2)	0.194 (4)
H109	0.0943	0.5069	0.2697	0.065*	0.194 (4)
C110	−0.0137 (18)	0.531 (6)	0.327 (2)	0.0593 (16)	0.194 (4)
H110	−0.0536	0.4314	0.2936	0.071*	0.194 (4)
C111	−0.0324 (15)	0.647 (5)	0.393 (2)	0.0610 (18)	0.194 (4)
H111	−0.0871	0.6415	0.4104	0.073*	0.194 (4)
S2	0.22671 (7)	1.02453 (14)	0.60821 (6)	0.0445 (3)	
C13	0.2293 (2)	1.2089 (5)	0.52711 (19)	0.0334 (7)	
C14	0.2588 (2)	1.3996 (5)	0.5637 (2)	0.0412 (8)	
H14	0.2646	1.5215	0.5304	0.049*	
C15	0.2795 (3)	1.3940 (6)	0.6571 (2)	0.0473 (9)	
H15	0.3014	1.5117	0.6932	0.057*	
C16	0.2647 (2)	1.2027 (6)	0.6895 (2)	0.0464 (9)	
H16	0.2745	1.172	0.7505	0.056*	
N1	0.24103 (19)	0.9760 (4)	0.40255 (16)	0.0352 (6)	
N2	0.2615 (2)	0.6596 (4)	0.34595 (18)	0.0414 (7)	
C1	0.3275 (2)	0.9754 (5)	0.39196 (19)	0.0357 (8)	
C2	0.3389 (2)	0.7765 (5)	0.3565 (2)	0.0397 (8)	
C3	0.4206 (3)	0.7300 (6)	0.3371 (2)	0.0485 (10)	
H3	0.4304	0.597	0.3133	0.058*	
C4	0.4859 (3)	0.8794 (6)	0.3528 (2)	0.0482 (9)	
H4	0.5416	0.8495	0.3398	0.058*	0.969 (2)
C5	0.4722 (2)	1.0756 (6)	0.3878 (2)	0.0438 (9)	
H5'	0.5192	1.1755	0.3979	0.053*	0.031 (2)
C6	0.3929 (2)	1.1299 (6)	0.4083 (2)	0.0397 (8)	
H6	0.3838	1.2635	0.4319	0.048*	
C7	0.2048 (2)	0.7813 (5)	0.3739 (2)	0.0381 (8)	
C12	0.1983 (2)	1.1614 (5)	0.4306 (2)	0.0368 (8)	
H12A	0.1332	1.139	0.4162	0.044*	
H12B	0.2104	1.285	0.3967	0.044*	

### Atomic displacement parameters (Å^2^)

**Table:**

	*U*^11^	*U*^22^	*U*^33^	*U*^12^	*U*^13^	*U*^23^
Cl1	0.0502 (6)	0.0575 (7)	0.0627 (7)	0.0008 (5)	0.0178 (5)	0.0148 (5)
Cl01	0.0502 (6)	0.0575 (7)	0.0627 (7)	0.0008 (5)	0.0178 (5)	0.0148 (5)
S1	0.0640 (9)	0.0371 (9)	0.0530 (9)	0.0065 (6)	0.0038 (7)	−0.0067 (6)
C8	0.055 (2)	0.0272 (18)	0.0366 (19)	0.0077 (16)	0.0083 (16)	0.0024 (14)
C9	0.052 (3)	0.031 (4)	0.084 (5)	−0.002 (3)	0.024 (3)	−0.008 (3)
C10	0.049 (3)	0.058 (4)	0.071 (4)	0.002 (3)	0.016 (3)	−0.002 (3)
C11	0.056 (4)	0.044 (3)	0.078 (4)	0.007 (3)	0.005 (3)	−0.005 (3)
S101	0.0640 (9)	0.0371 (9)	0.0530 (9)	0.0065 (6)	0.0038 (7)	−0.0067 (6)
C108	0.055 (2)	0.0272 (18)	0.0366 (19)	0.0077 (16)	0.0083 (16)	0.0024 (14)
C109	0.052 (3)	0.031 (4)	0.084 (5)	−0.002 (3)	0.024 (3)	−0.008 (3)
C110	0.049 (3)	0.058 (4)	0.071 (4)	0.002 (3)	0.016 (3)	−0.002 (3)
C111	0.056 (4)	0.044 (3)	0.078 (4)	0.007 (3)	0.005 (3)	−0.005 (3)
S2	0.0658 (6)	0.0309 (5)	0.0383 (5)	0.0023 (4)	0.0152 (4)	0.0043 (4)
C13	0.0431 (18)	0.0285 (18)	0.0311 (16)	0.0049 (14)	0.0140 (14)	0.0016 (13)
C14	0.055 (2)	0.035 (2)	0.0352 (18)	−0.0048 (16)	0.0134 (16)	0.0021 (15)
C15	0.066 (2)	0.038 (2)	0.0389 (19)	−0.0094 (18)	0.0140 (17)	−0.0089 (16)
C16	0.056 (2)	0.052 (2)	0.0334 (18)	−0.0029 (18)	0.0134 (16)	0.0007 (16)
N1	0.0522 (17)	0.0255 (15)	0.0290 (14)	0.0074 (12)	0.0120 (12)	0.0007 (11)
N2	0.0614 (19)	0.0277 (15)	0.0383 (15)	0.0089 (14)	0.0182 (14)	−0.0002 (12)
C1	0.051 (2)	0.0329 (19)	0.0247 (16)	0.0087 (15)	0.0121 (14)	0.0065 (13)
C2	0.060 (2)	0.0323 (19)	0.0287 (17)	0.0121 (16)	0.0151 (16)	0.0060 (14)
C3	0.069 (3)	0.040 (2)	0.042 (2)	0.0144 (19)	0.0252 (19)	0.0075 (16)
C4	0.055 (2)	0.051 (2)	0.044 (2)	0.0177 (19)	0.0223 (17)	0.0109 (17)
C5	0.051 (2)	0.046 (2)	0.0360 (18)	0.0053 (17)	0.0137 (16)	0.0125 (16)
C6	0.056 (2)	0.0347 (19)	0.0305 (17)	0.0082 (17)	0.0137 (15)	0.0050 (14)
C7	0.057 (2)	0.0284 (19)	0.0284 (16)	0.0057 (16)	0.0098 (15)	0.0040 (13)
C12	0.0502 (19)	0.0261 (17)	0.0349 (17)	0.0099 (15)	0.0118 (15)	0.0019 (14)

### Geometric parameters (Å, º)

**Table:**

Cl1—C5	1.750 (4)	C13—C12	1.502 (4)
Cl01—C4	1.740 (5)	C14—C15	1.419 (5)
S1—C11	1.724 (6)	C14—H14	0.95
S1—C8	1.740 (7)	C15—C16	1.357 (5)
C8—C9	1.389 (9)	C15—H15	0.95
C8—C7	1.468 (6)	C16—H16	0.95
C9—C10	1.396 (9)	N1—C1	1.386 (4)
C9—H9	0.95	N1—C7	1.388 (4)
C10—C11	1.337 (7)	N1—C12	1.468 (4)
C10—H10	0.95	N2—C7	1.319 (4)
C11—H11	0.95	N2—C2	1.385 (5)
S101—C108	1.707 (18)	C1—C6	1.389 (5)
S101—C111	1.717 (17)	C1—C2	1.408 (5)
C108—C109	1.351 (17)	C2—C3	1.399 (5)
C108—C7	1.411 (15)	C3—C4	1.366 (5)
C109—C110	1.401 (17)	C3—H3	0.95
C109—H109	0.95	C4—C5	1.398 (5)
C110—C111	1.352 (16)	C4—H4	0.95
C110—H110	0.95	C5—C6	1.383 (5)
C111—H111	0.95	C5—H5'	0.95
S2—C16	1.700 (4)	C6—H6	0.95
S2—C13	1.734 (3)	C12—H12A	0.99
C13—C14	1.372 (5)	C12—H12B	0.99
			
C11—S1—C8	91.4 (3)	S2—C16—H16	123.9
C9—C8—C7	131.8 (6)	C1—N1—C7	106.6 (3)
C9—C8—S1	109.5 (4)	C1—N1—C12	123.5 (3)
C7—C8—S1	118.3 (4)	C7—N1—C12	129.6 (3)
C8—C9—C10	113.1 (7)	C7—N2—C2	105.6 (3)
C8—C9—H9	123.4	N1—C1—C6	131.1 (3)
C10—C9—H9	123.4	N1—C1—C2	105.2 (3)
C11—C10—C9	113.6 (6)	C6—C1—C2	123.7 (3)
C11—C10—H10	123.2	N2—C2—C3	131.3 (3)
C9—C10—H10	123.2	N2—C2—C1	110.1 (3)
C10—C11—S1	112.0 (5)	C3—C2—C1	118.6 (3)
C10—C11—H11	124.0	C4—C3—C2	118.8 (3)
S1—C11—H11	124.0	C4—C3—H3	120.6
C108—S101—C111	91.5 (11)	C2—C3—H3	120.6
C109—C108—C7	124.7 (18)	C3—C4—C5	121.2 (3)
C109—C108—S101	111.3 (12)	C3—C4—Cl01	117.4 (12)
C7—C108—S101	123.9 (16)	C5—C4—Cl01	121.4 (12)
C108—C109—C110	113.3 (16)	C3—C4—H4	119.4
C108—C109—H109	123.4	C5—C4—H4	119.4
C110—C109—H109	123.4	C6—C5—C4	122.5 (3)
C111—C110—C109	112.5 (19)	C6—C5—Cl1	118.5 (3)
C111—C110—H110	123.7	C4—C5—Cl1	118.9 (3)
C109—C110—H110	123.7	C6—C5—H5'	118.7
C110—C111—S101	111.4 (17)	C4—C5—H5'	118.7
C110—C111—H111	124.3	C5—C6—C1	115.3 (3)
S101—C111—H111	124.3	C5—C6—H6	122.3
C16—S2—C13	91.85 (17)	C1—C6—H6	122.3
C14—C13—C12	126.3 (3)	N2—C7—N1	112.4 (3)
C14—C13—S2	110.8 (2)	N2—C7—C108	122.2 (13)
C12—C13—S2	122.8 (2)	N1—C7—C108	125.3 (14)
C13—C14—C15	112.3 (3)	N2—C7—C8	123.3 (4)
C13—C14—H14	123.9	N1—C7—C8	124.3 (4)
C15—C14—H14	123.9	N1—C12—C13	113.7 (3)
C16—C15—C14	112.9 (3)	N1—C12—H12A	108.8
C16—C15—H15	123.6	C13—C12—H12A	108.8
C14—C15—H15	123.6	N1—C12—H12B	108.8
C15—C16—S2	112.2 (3)	C13—C12—H12B	108.8
C15—C16—H16	123.9	H12A—C12—H12B	107.7
			
C11—S1—C8—C9	3.5 (8)	C2—C3—C4—Cl01	−179.3 (12)
C11—S1—C8—C7	177.2 (8)	C3—C4—C5—C6	0.0 (5)
C7—C8—C9—C10	−177.9 (10)	Cl01—C4—C5—C6	179.4 (13)
S1—C8—C9—C10	−5.3 (11)	C3—C4—C5—Cl1	−179.8 (3)
C8—C9—C10—C11	4.8 (9)	Cl01—C4—C5—Cl1	−0.4 (13)
C9—C10—C11—S1	−2.0 (6)	C4—C5—C6—C1	0.2 (5)
C8—S1—C11—C10	−0.9 (6)	Cl1—C5—C6—C1	180.0 (2)
C111—S101—C108—C109	0 (3)	N1—C1—C6—C5	−178.5 (3)
C111—S101—C108—C7	−177 (4)	C2—C1—C6—C5	−0.4 (5)
C7—C108—C109—C110	177 (4)	C2—N2—C7—N1	0.2 (4)
S101—C108—C109—C110	0 (4)	C2—N2—C7—C108	177 (2)
C108—C109—C110—C111	1 (4)	C2—N2—C7—C8	179.1 (6)
C109—C110—C111—S101	−1.2 (17)	C1—N1—C7—N2	−0.2 (3)
C108—S101—C111—C110	0.9 (16)	C12—N1—C7—N2	173.8 (3)
C16—S2—C13—C14	0.0 (3)	C1—N1—C7—C108	−177 (2)
C16—S2—C13—C12	−177.1 (3)	C12—N1—C7—C108	−3 (2)
C12—C13—C14—C15	177.4 (3)	C1—N1—C7—C8	−179.1 (6)
S2—C13—C14—C15	0.4 (4)	C12—N1—C7—C8	−5.1 (7)
C13—C14—C15—C16	−0.7 (5)	C109—C108—C7—N2	−24 (5)
C14—C15—C16—S2	0.7 (4)	S101—C108—C7—N2	152 (2)
C13—S2—C16—C15	−0.4 (3)	C109—C108—C7—N1	153 (3)
C7—N1—C1—C6	178.5 (3)	S101—C108—C7—N1	−31 (5)
C12—N1—C1—C6	4.1 (5)	C109—C108—C7—C8	−150..90
C7—N1—C1—C2	0.2 (3)	S101—C108—C7—C8	20..80
C12—N1—C1—C2	−174.3 (3)	C9—C8—C7—N2	142.3 (10)
C7—N2—C2—C3	−179.0 (3)	S1—C8—C7—N2	−29.7 (11)
C7—N2—C2—C1	−0.1 (4)	C9—C8—C7—N1	−39.0 (15)
N1—C1—C2—N2	−0.1 (3)	S1—C8—C7—N1	149.0 (5)
C6—C1—C2—N2	−178.6 (3)	C9—C8—C7—C108	−170..80
N1—C1—C2—C3	179.0 (3)	S1—C8—C7—C108	20..80
C6—C1—C2—C3	0.5 (5)	C1—N1—C12—C13	−75.6 (4)
N2—C2—C3—C4	178.5 (3)	C7—N1—C12—C13	111.3 (4)
C1—C2—C3—C4	−0.3 (5)	C14—C13—C12—N1	129.4 (3)
C2—C3—C4—C5	0.0 (5)	S2—C13—C12—N1	−54.0 (4)

### Hydrogen-bond geometry (Å, º)

**Table:**

*D*—H···*A*	*D*—H	H···*A*	*D*···*A*	*D*—H···*A*
C16—H16···N2^i^	0.95	2.62	3.369 (4)	136
C12—H12*B*···N2^ii^	0.99	2.69	3.654 (4)	165
C12—H12*B*···S1^ii^	0.99	2.99	3.599 (4)	120

Symmetry codes: (i) *x*, −*y*+3/2, *z*+1/2; (ii) *x*, *y*+1, *z*.
